# A national cohort study of parental socioeconomic status and non-fatal suicidal behaviour-the mediating role of school performance

**DOI:** 10.1186/1471-2458-12-17

**Published:** 2012-01-09

**Authors:** Beata Jablonska, Frank Lindblad, Viveca Östberg, Lene Lindberg, Finn Rasmussen, Anders Hjern

**Affiliations:** 1 Division of Applied Public Health, Department of Public Health Sciences, Karolinska Institutet, Stockholm, Sweden; 2 Department of Neuroscience, Child and Adolescent Psychiatry, Uppsala University, Uppsala, Sweden; 3 Stress Research Institute, Stockholm University, Stockholm, Sweden; 4 Centre for Health Equity Studies (CHESS), Stockholm University/Karolinska Institutet, Stockholm, Sweden; 5 Child and Adolescent Public Health Epidemiology Group, Department of Public Health Sciences, Karolinska Institutet, Stockholm, Sweden; 6 Department of Children's and Women's health, Uppsala University, Uppsala, Sweden

**Keywords:** Non-fatal suicidal behaviour, Socioeconomic status, School performance, Cohort studies

## Abstract

**Background:**

A link between low parental socioeconomic status and mental health problems in offspring is well established in previous research. The mechanisms that explain this link are largely unknown. The present study investigated whether school performance was a mediating and/or moderating factor in the path between parental socioeconomic status and the risk of hospital admission for non-fatal suicidal behaviour.

**Methods:**

A national cohort of 447 929 children born during 1973-1977 was followed prospectively in the National Patient Discharge Register from the end of their ninth and final year of compulsory school until 2001. Multivariate Cox proportional hazards and linear regression analyses were performed to test whether the association between parental socioeconomic status and non-fatal suicidal behaviour was mediated or moderated by school performance.

**Results:**

The results of a series of multiple regression analyses, adjusted for demographic variables, revealed that school performance was as an important mediator in the relationship between parental socioeconomic status and risk of non-fatal suicidal behaviour, accounting for 60% of the variance. The hypothesized moderation of parental socioeconomic status-non-fatal suicidal behaviour relationship by school performance was not supported.

**Conclusions:**

School performance is an important mediator through which parental socioeconomic status translates into a risk for non-fatal suicidal behaviour. Prevention efforts aimed to reduce socioeconomic inequalities in non-fatal suicidal behaviour among young people will need to consider socioeconomic inequalities in school performance.

## Background

It is well recognised, that children from families with low socioeconomic status (SES) suffer from poor health more often and are more likely to face a wide range of other adversities than their counterparts from families with high SES [1-3]. Early socioeconomic disadvantage may also have lasting health consequences throughout the course of their life [4-6]. Much less is known about the underlying processes through which childhood socioeconomic disadvantage translates into poor health later in life.

It has been a consistent finding that success in the school system depends heavily on the parents' SES [7-10]. Children from households with low SES do worse at school and achieve lower levels of education as adults than comparisons [11,12]. Educational outcomes, in turn, are critical to stratification processes affecting future employment opportunities and earnings potential [13]. Such stratification processes have been shown to have a strong influence on health outcomes. Better employment opportunities, safer work environments, and increased income, allowing investments in health and reducing economic instability, also enhance health and well-being. These work characteristics are, in turn, associated with a higher social position that is a predictor of good health per se [14]. Education is also associated with better health literacy and healthier lifestyles [15]. The benefits of education and the opportunities it provides are not confined to physical health but extend to mental health as well. A mounting body of literature indicates that education plays a major role in promoting mental health and preventing mental illness [16].

Despite this evidence, little emphasis has been placed on educational achievements during the school years as a possible link between low SES in childhood and the increased risk of poor mental health later in life. Although the hypothesis that the effect of childhood SES on subsequent mental health operates through its effect on education has been tested [4,17-19] and confirmed [18,19] in several studies, the research in this area has focused on the mediating role of adult educational attainment. The potentially cumulative effect of low parental SES and poor school performance also needs to be considered.

Suicidal behaviour among young people is one important mental health outcome where low parental SES, low intelligence (IQ) and poor school performance seem to increase risk [20-24]. In addition, findings indicate that rather than having a threshold effect, the risk of non-fatal suicidal behaviour (NFSB) in young people follows a socioeconomic and educational or IQ gradient [21,22,25,26]. Adolescence/young adulthood is a relatively healthy period, however the vulnerability to NFSB is most pronounced during this stage of life [27-29]. Moreover, NFSB rates among young people in Sweden have steadily increased over the past decade. In 2009, the rates of hospital admissions for NFSB in the 15-24 year-old group were 120 in every 100,000 men and 285 in every 100,000 women [29].

In conclusion, relatively limited attention has been given to the mechanisms that may bridge or modify the relationship between parental SES and mental health in young people. The aim of this register-based study is to investigate whether the association between childhood socioeconomic environment and NFSB in young people, an understudied age-group in this sense, is mediated or moderated by school performance.

## Results

A total of 4798 individuals were hospitalised due to NFSB at least once during the study period. The majority (81%) had been categorised as intentional and the remaining 19% as events of undetermined intent. The most frequent method of injury was poisoning-86% of the cases categorised as 'intentional injury' and 69% of the cases categorised as the 'event of undetermined intent' (Table 1).

**Table 1:** Methods of non-fatal suicidal ehavior (NFSB) in intentional and undetermined cases

	Intentional NFSB N (%)	Event of undetermined intent N (%)
Poisoning	3329 (85.8)	631 (68.8)

Poisoning by and exposure to alcohol	64 (1.6)	16 (1.7)

Sharp object	221 (5.7)	36 (3.9)

Hanging, strangulation, suffocation	43 (1.1)	-

Jumping from high place	57 (1.5)	-

Smoke, fire, flames, steam, hot vapours	16 (0.4)	101 (11.1)

Other/Unspecified means	151 (3.9)	133 (14.5)

All	3881	917

Descriptive data on NFSB and grade point average in relation to demographic data are presented in Table 2. There was a gradient between parental SES, rates of hospitalisation and grade point average: the lower the parental SES the higher the rate of hospitalisation and the lower the grade point average. Men had lower grade point average and hospitalisation rate than women. Young people of mixed ethnicity had a lower grade point average and higher hospitalisation rate than young people with a Swedish and non-Swedish background. For the residency category, both grade point average and hospitalisation rate were quite similar between the groups.

**Table 2:** Number of cases and crude rates of hospital admission due to non-fatal suicidal behaviour (NFSB) and mean of grade point average by parental SES and demographic factors

	Number of participants and distribution	Number of cases and crude rates of hospital admission due to NFSB			Grade point average
	**N (%)**	**All** **N (%)**	**Women** **N (%)**	**Men** **N (%)**	**Mean (SD)**

*Gender*					
Men	230 275 (51.4)	1649 (0.7)	-	-	3.11 (0.68)
Women	217 654 (48.6)	3149 (1.4)	-	-	3.38 (0.65)

*Parental SES*					
Higher level non-manuals	86 821 (19.4)	661 (0.8)	426 (1.0)	235 (0.5)	3.58 (0.61)
Middle level non-manuals	102 967 (23.0)	922 (0.9)	631 (1.3)	291 (0.5)	3.37 (0.62)
Lower level non-manuals	56 814 (12.7)	570 (1.0)	361 (1.3)	209 (0.7)	3.20 (0.64)
Skilled workers	72 217 (16.1)	844 (1.2)	554 (1.6)	290 (0.8)	3.04 (0.64)
Unskilled workers	63 808 (14.2)	948 (1.5)	626 (2.0)	322 (1.0)	2.93 (0.66)
Other	65 302 (14.6)	853 (1.3)	551 (1.7)	302 (0.9)	3.10 (0.67)

*Ethnicity*					
Swedish	391 067 (87.3)	3878 (1.0)	2534 (1.3)	1344 (0.7)	3.25 (0.67)
Non-Swedish	25 641 (5.7)	359 (1.4)	244 (2.1)	115 (1.0)	3.18 (0.68)
Mixed	31 221 (7.0)	561 (1.8)	377 (2.5)	184 (1.2)	3.19 (0.70)

*Residency*					
Stockholm, Malmö, Gothenburg	114 833 (25.6)	1341 (1.2)	903 (1.6)	438 (0.7)	3.31 (0.68)
Other city	233 697 (52.2)	2463 (1.1)	1573 (1.4)	890 (0.7)	3.23 (0.67)
Rural	99 346 (22.2)	993 (1.0)	672 (1.4)	321 (0.6)	3.19 (0.67)

Parental SES was associated with school performance [standardised beta coefficients ranged between -0.34 (children of unskilled workers had 0.34 units lower grade point average than children of higher-level non-manuals) and -0.13 (children of unskilled workers had 0.13 units lower grade point average than children of higher-level non-manuals); all at *p *< 0.0001], indicating that the condition required at the first step of mediation was fulfilled. The association between school performance and NFSB (HR = 0.39, 95% CI = 0.38-0.41) satisfied the requirements of the second step of mediation.

Table 3 presents Cox regression models of the mediation analysis of parental SES, school performance and hospital admission due to NFSB. In model 1 (step 3 of mediation), adjusted for year of birth and gender, ethnicity and residency, the risk of NFSB increased with decreasing level of parental SES. Additional adjustment for grade point average in model 2 (step 4 of mediation) abolished this increased risk. These patterns were similar for both men and women. The results from the Sobel test confirmed that school performance significantly mediated the association between parental SES and the risk of NFSB (*z *= -38.9, *p *< .0001), accounting for 60% of the explained variance.

**Table 3:** Hazard ratios (95% confidence intervals) for hospital admission due to non-fatal suicidal behaviour (NFSB) by parental SES

	All		Women		Men	
	Model 1 (HR, 95%CI)	Model 2 (HR, 95%CI)	Model 1 (HR, 95%CI)	Model 2 (HR, 95%CI)	Model 1 (HR, 95%CI)	Model 2 (HR, 95%CI)
*Parental SES*						
Higher level non-manuals	1†	1†	1†	1†	1†	1†
Middle level non-manuals	1.2 (1.1-1.3)	1.0 (0.9-1.1)	1.3 (1.1-1.4)	1.1 (0.9-1.2)	1.1 (0.9-1.2)	0.8 (0.7-1.0)
Lower level non-manuals	1.3 (1.2-1.5)	0.9 (0.8-1.0)	1.3 (1.1-1.5)	0.9 (0.8-1.1)	1.4 (1.1-1.7)	0.9 (0.8-1.1)
Skilled workers	1.5 (1.4-1.7)	0.9 (0.9-1.1)	1.6 (1.4-1.8)	1.0 (0.9-1.1)	1.5 (1.3-1.8)	0.9 (0.7-1.0)
Unskilled workers	1.9 (1.7-2.1)	1.0 (0.9-1.2)	1.9 (1.7-2.2)	1.1 (0.9-1.2)	1.9 (1.6-2.2)	0.9 (0.8-1.1)
Other	1.7 (1.6-1.9)	1.1 (1.0-1.2)	1.7 (1.5-1.9)	1.1 (1.0-1.3)	1.7 (1.5-2.1)	1.0 (0.9-1.2)

Model 1 = adjusted for year of birth, gender, ethnicity, and residency. Model 2 = as model 1 with additional adjustment for mean of grade point average

† = reference group

When interaction terms were add to the Cox proportional hazard regression model, the strength of the inverse association between parental SES and risk of NFSB did not vary according to school performance (grade point average). No significant interactions were found between the level of parental SES and school performance with respect to the effect of these variables on risk of NFSB (Table 4).

**Table 4:** Hazard ratios (95% confidence intervals) for hospital admission due to non-fatal suicidal behaviour NFSB by SES*Grade point average

	All (HR, 95% CI)	Women (HR, 95% CI)	Men (HR, 95% CI)
SES*Grade point average			
Higher level non-manuals*Grade point average	1†	1†	1†
Middle level non-manuals*Grade point average	1.0 (0.9-1.2)	1.0 (0.8-1.2)	0.9 (0.7-1.2)
Lower level non-manuals*Grade point average	1.1 (0.9-1.3)	1.1 (0.9-1.4)	1.0 (0.8-1.4)
Skilled workers*Grade point average	1.0 (0.8-1.1)	0.9 (0.8-1.1)	0.9 (0.7-1.2)
Unskilled workers*Grade point average	1.1 (0.9-1.3)	1.1 (0.9-1.3)	1.0 (0.8-1.3)
Other*Grade point average	0.9 (0.8-1.1)	0.8 (0.7-1.0)	1.1 (0.9-1.4)

Adjusted for year of birth, gender, ethnicity, SES, residency and mean of grade point average

† = reference group

## Discussion

The main finding of this study is that the negative association between parental SES and NFSB during adolescence/young adulthood to a large extent was mediated by school performance, accounting for as much as 60% of the variance. These findings are consistent with the large body of literature that has demonstrated the parallel links of parental SES to school performance [11,12] and NFSB, respectively [21]. However, this is the first study to examine the mediating role of school performance. We found no evidence for the hypothesis that the vulnerability to NFSB as a function of family socioeconomic environment may be moderated by school performance. This indicates that, although children with low SES face greater risk of poor school performance and, due to this fact, are overrepresented among self-injuring youth, the effect of poor school performance on the risk of NFSB seems to be equal for all SES-groups.

Parental SES had no effect on the risk of NFSB over and above what was mediated by school performance suggesting that childhood disadvantages act on a later risk of NFSB primarily in terms of the pathway effect. This means that low SES children tend to have a higher risk of NFSB and that risk can be understood in terms of them performing worse at school than comparisons. Thus, parental SES contributes to the intergenerational transmission of health disparities by placing children on different pathways leading to different mental health outcomes. The poor school performance of low SES children may be seen as a prolongation of the disadvantage experienced by one generation in the lives of the next and different mechanisms induced from conception onwards, by both material and non-material parental disadvantage, may be involved in impeding an underprivileged child's ability to reach his or her academic potential. For example, recent studies suggest that low SES increases the risk of exposure to adverse circumstances surrounding gestation and birth (e.g. inadequate nutrition, toxic exposure and stress) [30] that can negatively influence brain development [31,32] and its cognitive function in several ways [33]. Low parental SES is likely to be accompanied by financial strain and psychosocial adversity related to poor parental health, alcohol misuse, and family disruption [1,3]. These circumstances can undermine the amount and quality of stimulation that the child receives at home which is necessary for his/her optimal cognitive development [34]. Furthermore, it is more common for low-SES children to live in poor neighbourhoods and attend schools with high percentages of disadvantaged and low-achieving students [35]. These environmental characteristics have been shown to account for some of the risk of underperformance on an individual level, over and above the effect of individual SES and cognitive abilities [36].

Inter-generational disadvantage resulting in poor school performance and, in consequence in NFSB, can also arise as an effect of restricted access to human, social, and cultural resources that enhance educational outcomes. Parental education, which is correlated with SES [37], may play a particularly important role in this regard. Although low educated parents may value education as much as high educated parents, they often lack the ability to encourage their children to value education. Low-educated parents are less able to provide their children with qualified help with homework, early training in behaviours and skills (e.g. literacy) that are valued by schools, and to encourage them to achieve the expected outcomes [38-40]. As a result, low-SES children are less familiar with school culture, values and expectations and therefore less well equipped to achieve educational goals. Better-educated parents are also more informed regarding strategic educational choices and better equipped to communicate with teachers [3,8,41]. These characteristics allow the parent to closely monitor the child's performance and be proactive in preventing academic failure.

The differences in school performance between low and high-SES students may, to some extent, reflect inherited cognitive ability. There are, however, findings which indicate lower heritability of cognition among children from lower, rather than higher, SES backgrounds [42], thus assigning greater importance for their cognitive outcomes to environmental factors.

School performance may be an important path because it is highly determinative of other exposures related to mental health. Early school performance is an important source for identity formation and the development of social roles [43], for how peers rate each other's worth in the school's social hierarchy [44], and clearly contributes to educational attainment and occupational opportunities later in life. By enhancing cognitive abilities such as critical thinking, and problem solving, school performance may also directly influence mental health. These are useful not only within the confines of the classroom but they also apply to situations outside the realm of the school and result in *real-world *benefits, including better health [45].

The presence of full statistical mediation suggests that school performance provides the key to understanding the mechanism through which long-term effects of parental SES on the risk of self-injury was transmitted and contributes to growing evidence that school performance may play a key role in the prediction of NFSB. It also extends previous findings by suggesting that school performance is not only an independent predictor of NFSB but also serves as a mediator between parental SES and self-injury. These results are in line with previous findings showing that a large proportion of the effect of childhood SES on adult health operates through its effect on education [18,19]. However, whereas previous studies focused on educational attainment in adulthood, the current work suggests that educational stratification of mental health may already be discerned in the early stages of education.

The direct importance of school performance indicates that a child's SES is not fully determinative of later mental health problems expressed as NFSB. Thus, our findings suggest that when adequately meeting the academic needs of disadvantaged children, the negative health pathway caused by socioeconomic disadvantage may be counterbalanced or redirected.

### Limitations

The Hospital Discharge Register only includes NFSB cases admitted to in-patient care. It does not detect cases not seeking medical help after an act of NFSB or those who attended primary or specialized medical care, but were not admitted to in-patient care. Compared with a recent population-based health survey conducted in 2009 by the National Public Health Institute, the figures for NFSB are lower in the present study. In that survey, 8% of women and 3% of men aged 19-29, reported that they had, at least once, tried to take their own life [46]. It cannot be taken for granted that the role of socioeconomic inequality in school grades is similar in cases of NFSB not reported to the medical system or in less-severe cases not in need of in-patient care.

The register-based design of this study did not allow for the control of mental health conditions that do not lead to inpatient care. Thus, the reverse association, meaning that poor school performance may be a consequence of mental health problems, cannot be entirely excluded.

## Conclusions

This study demonstrated that a substantial part of the association between parental SES and NFSB could be ascribed to the intermediate effect of school performance. This suggests that school interventions adequately meeting the academic needs of children from low socioeconomic background may have the potential to counterbalance the negative health effects caused by socioeconomic disadvantage. Educational strategies directed toward low-SES students' academic outcomes should be accompanied by the social ones that aim to reduce socioeconomic differences in psychosocial and environmental factors implicated in the academic success. The potential role of school performance as a pathway between parental SES and other health outcomes should be investigated further in future studies.

The evidence from the present study also suggests that the effect of poor school performance on the risk of NFSB seems to be equal for all SES-groups. Thus, to strengthen prevention strategies relating to mental health, investment in educational support for students failing to meet their potential is needed in all socioeconomic groups. Whether the mechanisms linking poor school performance and the risk of mental health problems are distinct for different SES groups is worthy of further investigation.

## Methods

This study was based on data from national registers held by the Swedish National Board of Health and Welfare and Statistics Sweden. The key to these registers is the unique personal identification number. This number was used to link data from the registers to each person. The study was approved by the regional ethical review board at Karolinska Institutet, Stockholm, Sweden.

### Study population

The study population consisted of the entire Swedish population born between 1973 and 1977 (N = 491 258), registered as residents in the Swedish Population and Housing Census of 1985 and with reports in the first five birth cohorts in the National School Register (see below). Individuals who had been admitted to a hospital due to a psychiatric disorder and/or NFSB before finishing ninth grade (n = 1670) were excluded. On the scale ranging from a minimum of 1.0 to a maximum of 5.0, their grade point average was 2.84 (standard deviation (SD) = 0.68) which was similar to those who had been admitted to a hospital after compulsory school, 2.87 (SD = 0.73). Individuals who had three or more incomplete courses, i.e. courses that had not been formally graded by the end of the examination period due to an insufficient basis to evaluate student performance, (n = 7062) were also excluded because of their unreliable grade point average, as were foreign-born children (n = 34,597) because of the negative influence of migration on school performance [47]. In total, 447 929 persons comprised the study population.

### Outcome variable

The outcome variable-first-time hospital admission due to "*purposely self-inflicted poisoning or injury/suicide (attempted)*" (ICD-10)-was obtained through individual record linkage to the National Hospital Discharge Register from 1987 to 2001. Hospital admission involves staying at a hospital for at least one night. NFSB was defined according to the ninth revision of the World Health Organization (WHO) International Classification of Diseases (ICD-9) (intentional self-harm E950-959 and event of undetermined intent E980-989) during 1987-1996 and the tenth revision (ICD-10) (intentional self-harm X60-84 and event of undetermined intent Y10-34) during 1997-2001.

### School performance

Data on grade point averages at the time of leaving compulsory school (usually at 16 years of age) was collected from the National School Register. This register encompasses data from all public schools since 1988 and also data from all non-public schools, (less than 5% of all Swedish schools) since 1993. Until 1996 a five-graded relative scale was used in the Swedish school system, supervised by the Swedish School Authority through national tests in core subjects. The quality of the data in the National School Register is high and summary statistics are published regularly http://www.skolverket.se. Grade point average was calculated on the basis of the 17 school subjects and ranged from a minimum of 1.0 to a maximum of 5.0, mean = 3.2, SD = 0.7.

### Parental socioeconomic status

The socioeconomic status of the household was obtained from the Swedish Population and Housing Census of 1985. SES was defined according to the classification used by Statistics Sweden which is based on occupation and also takes the occupation's level of qualification, type of production and position of work of the head of the household into account. Six categories of SES were created: unskilled workers, skilled workers, lower-level non-manuals, middle-level non-manuals, higher-level non-manuals, and others (i.e. the self-employed, farmers, students, housewives, old age/sickness disability pensioners, long-term unemployed). The highly heterogeneous composition of the SES category "other" was due to the relatively small numbers of individuals belonging to each subcategory.

### Demographic variables

Demographic indicators were created through linkage to the Swedish Population and Housing Census of 1985: year of birth, gender, ethnicity, and geographical location of the home (residency). These variables have been selected because of their association to parental SES, school performance and NFSB in previous studies [21]. Information about parental country of birth was used to create a three-category proxy for ethnicity: Swedish (both parents born in Sweden), non-Swedish (neither parent born in Sweden), and mixed (one parent born in Sweden and one parent born in another country).

### Statistical methods

Multivariate analyses were performed by Cox proportional hazards regression of time to *first hospital admission due to NFSB *as the outcome variable. Time in the study was calculated with the entry date defined as the date of graduation and the exit date as the date of the first hospital admission, date of death from the National Cause of Death Register, date of emigration from the Register of the Total Population or the end of follow-up (December 2001).

The mediating and moderating role of school performance in the association between parental SES and NFSB was tested according to the recommendations of Baron and Kenny (1986) [48]. The criteria required for mediation to occur were as follows: 1) parental SES predicts school performance; 2) school performance predicts NFSB; 3) parental SES predicts NFSB; 4) the relationship between parental SES and NFSB is attenuated to non-significance but not absolute zero (in the case of partial mediation) or abolished (in the case of total mediation), when controlling for school performance. Linear regression with dummy variables was used to test whether the first criterion was satisfied and Cox proportional hazards regressions were used to verify satisfaction of all other criteria. Additionally, the Sobel test was used to assess the statistical reliability of the degree of mediation [48,49]. The amount of explained variance accounted for by the mediation was also calculated.

The interaction term between parental SES and grade point average was formed by multiplying dummy coded parental SES by grade point average. Cox proportional hazards regression was performed using parental SES, grade point average and the interaction term between parental SES and grade point average. To reduce potential problems with multicollinearity between the interaction term with its component variables in the analysis of moderating effect, school performance was mean-centered by subtracting its sample mean from all individuals' values, thus producing a revised sample mean of zero [50].

All models were simultaneously adjusted for potential confounding factors (year of birth, gender, ethnicity, and residency). Year of birth was entered as a continuous variable into the regression models in accordance with the linear relation of this variable to the outcome. Other socio-demographic variables were entered as categorical variables into the models, and when necessary with the use of dummy variables. The SPSS software package, version 17.0 was used in all statistical analyses.

## Competing interests

The authors declare that they have no competing interests.

## Authors' contributions

BJ, FL, AH conceived of the study, participated in its design and drafted the manuscript. FR, AH participated in the acquisition of data. BJ performed the statistical analysis and participated in interpretation of data. VÖ helped to draft the manuscript. FL, VÖ, LL, FR, AH contributed to interpretation of the results and revision of the manuscript. All authors read and approved the final manuscript.

## Pre-publication history

The pre-publication history for this paper can be accessed here:

http://www.biomedcentral.com/1471-2458/12/17/prepub

## References

[B1] Halldorsson M Kunst AE Köhler L Mackenbach JP Socioeconomic inequalities in the health of children and adolescents. A comparative study of the five Nordic countries Eur J Public Health 2001 10 281 288

[B2] McMunn AM Nazroo JY Marmot MG Boreham R Goodman R Children's emotional and behavioural well-being and the family environment: findings from the Health Survey for England Soc Sci Med 2001 53 423 440 10.1016/S0277-9536(00)00346-4 11459394

[B3] Power C Matthews S Origins of health inequalities in a national population sample Lancet 1997 350 1584 1589 10.1016/S0140-6736(97)07474-6 9393337

[B4] Huurre T Aro H Rahkonen O Well-being and health behaviour by parental socioeconomic status: a follow-up study of adolescents aged 16 until age 32 years Soc Psychiatry Psychiatr Epidemiol 2003 38 249 255 10.1007/s00127-003-0630-7 12719840

[B5] Weitoft GR Hjern A Haglund B Rosen M Mortality, severe morbidity, and injury in children living with single parents in Sweden: a population-based study Lancet 2003 361 289 295 10.1016/S0140-6736(03)12324-0 12559862

[B6] Power C Stansfeld SA Matthews S Manor O Hope S Childhood and adulthood risk factors for socio-economic differentials in psychological distress: evidence from the 1958 British birth cohort Soc Sci Med 2002 55 1989 2004 10.1016/S0277-9536(01)00325-2 12406466

[B7] Erikson R Jonson J Can Education be Equalized. The Swedish Case in Comparative Perspective 1996 New York: Westview Press

[B8] Jonsson JO Jansson S, Östberg V Utbildning som resurs under skoltiden och för framtiden: uppväxtfamilj och skolgång (Education as a resource in schooling and for the future: family and schooling) Barn och ungdomars välfärd 2001 SOU 2001:59. Stockholm: Fritzes 209 238

[B9] Gutman L Sameroff A Cole R Academic growth curve trajectories from 1st grade to 12th grade: effects of multiple social risk factors and preschool child factors Dev Psychol 2003 39 777 790 12859129 10.1037/0012-1649.39.4.777

[B10] Sirin SR Socioeconomic status and academic achievement: a meta-analytic review of research Rev Educ Res 2005 75 417 453 10.3102/00346543075003417

[B11] Bradley RH Corwyn RF Socioeconomic status and child development Annu Rev Psychol 2002 53 371 399 10.1146/annurev.psych.53.100901.135233 11752490

[B12] Lee V Burkam D Inequality at the Starting Gate: Social Background Differences in Achievement as Children Begin School 2002 Washington: Economic Policy Institute

[B13] Currie J Duncan T Early test scores, school quality and SES: long run effects on wage and employment outcomes Res Labor E 2001 20 103 132

[B14] Marmot M The Status Syndrome 2004 New York: Henry Holt

[B15] Wardle J Steptoe A Socioeconomic differences in attitudes and beliefs about healthy lifestyles J Epidemiol Community Health 2003 57 440 443 10.1136/jech.57.6.440 12775791 PMC1732468

[B16] Dalgard OS Mykletun A Rognerud M Johansen R Zahl PH Education, sense of mastery and mental health: results from a nationwide health monitoring study in Norway BMC Psychiatry 2007 7 20 10.1186/1471-244X-7-20 17519014 PMC1887526

[B17] Kestilä L Koskinen S Martelin T Rahkonen O Pensola T Aro H Aromaa A Determinants of health in early adulthood: what is the role of parental education, childhood adversities and own education? Eur J Public Health 2006 16 306 315 16141301 10.1093/eurpub/cki164

[B18] Der G Batty GD Deary IJ The association between IQ in adolescence and a range of health outcomes at 40 in the 19979 US National Longitudinal Study of Youth Intelligence 2009 3 573 580 10.1016/j.intell.2008.12.002 PMC2772900 19907663

[B19] McKenzie SK Carter K Blakely T Collings S The association of childhood socioeconomic position and psychological distress in adulthood: is it mediated by adult socioeconomic position? Longit Life Course Stud 2010 1 339 358

[B20] Fergusson DM Woodward LJ Horwod LJ Risk factors and life processes associated with the onset of suicidal behaviour during adolescence and early adulthood Psychol Med 2000 30 23 39 10.1017/S003329179900135X 10722173

[B21] Jablonska B Lindberg L Lindblad F Rasmussen F Östberg V Hjern A School performance and hospital admissions due to self-inflicted injury: a Swedish national cohort study Int J Epidemiol 2009 38 1334 1341 10.1093/ije/dyp236 19556329

[B22] Björkenstam C Weitoft GR Hjern A Nordström P Hallqvist J Ljung R School grades, parental education and suicide-a national register-based cohort study J Epidemiol Community Health 2011 65 993 998 10.1136/jech.2010.117226 20961868

[B23] Andersson L Allebeck P Gustafsson JE Association of IQ scores and school achievement with suicide in a 40-year follow-up of a Swedish cohort Acta Psychiatr Scand 2008 118 99e105 10.1111/j.1600-0447.2008.01171.x 18331576

[B24] Gunnell D Löfving S Gustaffson JE Allebeck P School performance and risk of suicide in early adulthood: follow-up of two national cohorts of Swedish schoolchildren J Affect Disord 2011 131 104 112 10.1016/j.jad.2011.01.002 21296426

[B25] Lyons RA Jones SJ Deacon T Heaven M Socioeconomic variation in injury in children and older people: a population based study Inj Prev 2003 9 33 37 10.1136/ip.9.1.33 12642556 PMC1730918

[B26] Batty GD Whitley E Deary IJ Gale CR Tynelius P Rasmussen F Psychosis alters association between IQ and future risk of attempted suicide: cohort study of 1, 109, 475 Swedish men BMJ 2010 340 c2506 10.1136/bmj.c2506 20522657 PMC2881197

[B27] Schmidtke A Bille-Brache U DeLeo D Attempted suicide in Europe: rates, trends and sociodemographic characteristics of suicide attempters during the period 1989-1992. Results of the WHO/EURO Multicentre Study on Parasuicide Acta Psychiatr Scand 1996 93 327 338 10.1111/j.1600-0447.1996.tb10656.x 8792901

[B28] Nock MK Borges G Bromet EJ Cha CB Kessler RC Sing Lee S Suicide and suicidal behavior Epidemiol Rev 2008 30 133 154 10.1093/epirev/mxn002 18653727 PMC2576496

[B29] The National Board of Health and Welfare Skador och förgiftningar behandlade i sluten vård Hospitalisation due to injuries and poisoning in Sweden 2009 2010 http://www.socialstyrelsen.se

[B30] Dawson G Ashman SB Carver LJ The role of early experience in shaping behavioral and brain development and its implications for social policy Dev Psychopathol 2000 12 695 712 10.1017/S0954579400004089 11202040

[B31] Hackman DA Farah MJ Socioeconomic status and the developing brain Trends Cogn Sci 2009 13 65 73 10.1016/j.tics.2008.11.003 19135405 PMC3575682

[B32] Tomalski P Johnson H The effects of early adversity on the adult and developing brain Curr Opin Psychiatry 2010 23 233 238 10.1097/YCO.0b013e3283387a8c 20308900

[B33] Hack M Breslau N Weissman B Aram D Klein N Borawski E Effect of very low birth weight and subnormal head size on cognitive abilities at school age N Engl J Med 1991 325 231 237 10.1056/NEJM199107253250403 2057024

[B34] Stein A Malmberg L-E Sylva K Barnes J Leach P the FCCC team The influence of maternal depression, caregiving, and socioeconomic status in the post-natal year on children's language development Child Care Health Dev 2008 34 603 612 10.1111/j.1365-2214.2008.00837.x 18549438

[B35] Statistics Sweden Barn, boendesegregation och skolresultat Children, segregated housing and school results 2007 Demografiska Rapporter (Demographic reports) 2

[B36] Gutman LM Feinstein L Children's Well-Being in Primary School: Pupil and School Effects 2008 London: Centre for Research on the Wider Benefits of Learning Institute of Education22235480

[B37] Statistics Sweden Utbildningsnivå efter indikator, socioekonomisk grupp och kön Level of education by the indicator, socioeconomic group and gender http://www.ssd.scb.se

[B38] Guo G Harris KM The mechanisms mediating the effects of poverty on children's intellectual development Demography 2000 37 431 447 10.1353/dem.2000.0005 11086569

[B39] De Civita M Pagani L Vitaro F Tremblay RE The role of maternal educational aspirations in mediating the risk of income source on academic failure in children from persistently poor families Child Youth Serv Rev 2004 26 749 769 10.1016/j.childyouth.2004.02.019

[B40] Werfhorst HG Hofstede S Cultural capital or relative risk aversion? Two mechanisms for educational inequality compared Br J Sociol 2007 58 391 415 10.1111/j.1468-4446.2007.00157.x 17727500

[B41] Due P Lynch J Holstein B Modvig J Socioeconomic health inequalities among a nationally representative sample of Danish adolescents: the role of different types of social relations J Epidemiol Community Health 2003 57 692 698 10.1136/jech.57.9.692 12933775 PMC1732594

[B42] Turkheimer E Haley A Waldron M D'Onofrio B Gottesman II Socioeconomic status modifies heritability of IQ in young children Psychol Sci 2003 14 623 628 10.1046/j.0956-7976.2003.psci_1475.x 14629696

[B43] Modin B Ostberg V Almquist Y Childhood peer status and adult susceptibility to anxiety and depression. A 30-year hospital follow-up J Abnorm Child Psychol 2010 39 187 199 10.1007/s10802-010-9462-6 20886279

[B44] Baumeister RF Campbell JD Krueger JI Vohs KD Does high self-esteem cause better performance, interpersonal success, happiness, or healthier lifestyles? Psychol Sci Public Interest 2003 4 1 44 10.1111/1529-1006.01431 26151640

[B45] Reynolds JR Ross CE Social stratification and health: education's benefit beyond economic status and social origins Soc Probl 1998 45 221 247 10.1525/sp.1998.45.2.03x0167k

[B46] Clevenpalm J Karlsson A-S Health On Equal Terms? Results from the 2006 Swedish National Public Health Survey 2009 Public Health: Swedish National Institute of

[B47] The Swedish National Agency for Education Vad påverkar resultaten i svensk grundskola? Kunskapsöversikt om betydelsen av olika faktorer Which factors affect the results of Swedish comprehensive school? An overview of the importance of different factors 2009 Stockholm: Fritzes

[B48] Baron RM Kenny DA The moderator-mediator variable distinction in social psychological research: conceptual, strategic, and statistical considerations J Pers Soc Psychol 1986 51 1173 1182 3806354 10.1037//0022-3514.51.6.1173

[B49] Herr NR Mediation with dichotomous outcomes http://nrherr.bol.ucla.edu/Mediation/logmed.html

[B50] Aiken LS West SG Multiple Regression: Testing and Interpreting Interactions 1991 Newbury Park: Sage

